# The effect of a 10-week TOCA Football System intervention program on sport-specific motor skills among junior footballers

**DOI:** 10.3389/fspor.2024.1339768

**Published:** 2024-05-13

**Authors:** Zoltán Tamás Szabó, Evelin Derkács, Balázs Deli, Viktória Prémusz, Lívia Vass, Henriette Pusztafalvi, Pongrác Ács

**Affiliations:** ^1^ Faculty of Health Sciences, Institute of Physiotherapy and Sport Science, University of Pécs, Pécs, Hungary; ^2^ Faculty of Health Sciences, Doctoral School of Health Sciences, University of Pécs, Pécs, Hungary; ^3^ Physical Activity Research Group, Szentágothai Research Centre, University of Pécs, Pécs, Hungary; ^4^ Faculty of Natural Sciences, Institute of Sport Science and Physical Education, University of Pécs, Pécs, Hungary; ^5^ Faculty of Health Sciences, Institute of Health Insurance, University of Pécs, Pécs, Hungary

**Keywords:** intervention study, junior football, TOCA Football System, motor skills, performance

## Abstract

**Introduction:**

The objective of our study was to examine, in addition to using the TOCA Football System tool and training method, the effect of a 10-week intervention on elite youth athletes in terms of their sport-specific motor skills and anthropometric variables.

**Methods:**

The study covered a group of 32 young players practicing football (U14) (13.45 ± 0.64 years). The junior U14 footballers were randomly assigned to an intervention or TOCA group (TG, *N* = 15, 13.25 ± 0.58 years) and a control group (CG, *N* = 17, 13.63 ± 0.66 years). Before starting the test, we performed full anthropometric measurements and assessed the sample's agility with and without the ball and their sport-specific endurance. The measurements were then repeated after the 10-week intervention.

**Results:**

Within-group analysis showed significant improvements in muscle mass (*p* < 0.001), sport-specific endurance (*p* < 0.001), (*p* < 0.004) and agility (in TG) both with and without the ball (*p* = 0.002), (*p* = 0.004) however, we did not find a significant change in body fat percentage in either group (*p* = 0.988, *p* = 0.288). In the CG, “agility with the ball” changed significantly only (*p* = 0.023). In the between-group analysis with a repeated-measures analysis of variance (mixed-design ANOVA), there was no significant interaction in any performance variables. The main findings of this study indicate that a TOCA Football training program in addition to normal training during the in-season period does not produce additional effects in anthropometric factors, sport-specific endurance and agility performance with the ball (dribbling) and without the ball in comparison with the control condition.

**Discussion:**

From a practical point of view, the presented anthropometric and physical profiles of players can be useful for football coaches in optimizing soccer training. Overall, it also can be concluded that the device can be safely used in the sensitive age group in terms of the development of motor skills since we did not find any negative effects during the use of the device in terms of the parameters we examined. In addition to the expansion of the number of elements and the inclusion of other age groups, it is advisable to carry out further complex tests, as the TOCA Football System offers many research opportunities.

## 1 Introduction

Football is the most popular sport and is played all over the world (1). In today's top football, the game is becoming faster and more physical, which demands exceptional skills from the players (2). According to Reilly et al. (3) and Davies (4), the modern game is primarily defined by power and speed. One-touch football, characteristic of modern football (5, 6), requires energy-demanding high-intensity movements from athletes (7, 8). As all elements of the game sped up, the sport-specific endurance (which means adapting to a large number of accelerations, decelerations and changes of direction and often repeated sprints) of the players also had to adapt to the increased expectations (9–12). If we add to this the number of matches held annually, which is approximately 45–55 on which they cover a total of 9–14 km on average, with high-intensity running accounting for 5%–15% of this distance, then we can conclude that the annual running volume of outstanding players at the international level requires amazing physical preparation. Due to the increased physical demands, athletes must have outstanding sport-specific skills (13–16). Based on a range of physical and biological characteristics (such as body mass, body composition, speed, agility, vertical jumping, power, repeated sprint ability, and endurance), researchers have differentiated more successful elite youth football players from those who were less successful at multiple age groups from U9-U21 (17–20). Therefore, in the world of football, more and more emphasis is being placed on training with a scientific approach.

The relationship between football and sports science began to strengthen approximately in the second half of the 20th century. By the 1980s, it became obvious that football could not rely on the traditional methods used in the previous decades in the long term. The effectiveness of scientific methods was proven by the fact that those clubs that changed their previous methods were more successful than those that stayed with the traditional methods used before (4). The early recognition of innovations and new training methods that appear in the sport can quickly give teams that apply them a step ahead. By the 2000s, the majority of elite Hungarian clubs founded their own academies. In recent years, further major great changes have taken place in Hungarian football, the economic environment has changed, which meant completely new funding through the introduction of the TAO support system. The aim of the operation was to prepare talented young footballers for professional football more effectively (17–19). According to international literature, these training workshops have become the most important talent development institutions in football (20). According to Rábai (18), the academic environment provides outstanding personal and infrastructural training opportunities for young footballers to become professional athletes. Among other things, this academic system enables the TOCA Football System to play a role in the preparation of young athletes. In Hungary, football academies are youth training institutions whose primary goal is to educate highly qualified and internationally competitive players (19). Currently, 19 such academies operate in Hungary, which work according to the operating conditions system used by the European Football Association (UEFA) by providing learning, housing, and professional and institutional conditions. In Hungary, the system of football academies is used primarily for the training and complex talent management of the sport, as well as the development of the careers of young footballers and the sport at the same time (21). Since the system has excellent conditions for selection and talent management (22), in recent years, academies have become prominent actors in the training of Hungarian youth (23). Despite all this, only a few of our young footballers make it to the best in Europe (24).

Thus, the methods, tools and tasks that promote the development of different physical and technical skills during football training should be given priority (25). The effect of the TOCA Football System has not been examined in this regard before. It can also be said that there are very few publications in English related to Hungarian football youth training methods. Given that its use is widespread in Hungary and many countries worldwide and that it is also readily used when providing training for junior footballers, the examination of the tool is of prime importance (26). Furthermore, sport for the health of young people is of prime importance in professional sport is also a strategic area of priority (27, 28). The novelty of the TOCA Football system lies in the fact that the sport-specific technical elements (first touch, passing, stops and starts, change of directions, etc.)—that form the basis of the technical toolkit of a footballer—can be practiced by the athlete with a higher repetition rate than it would be possible with traditional training methods (26). By changing the physical load in this way, the sport-specific technical elements can become a muscle memory action faster (29, 30). One of the most important goals of the various objective tests known and recognized by international research is to be able to monitor the effectiveness of the training and the defined physiological objective with the established periodization (31). Extensive examination of junior footballers can range from human biological values through lower-limb muscle strength and running speed to spiroergometric measurements. It evaluates the players in a complex manner (32), and its application is essential to create the possibility of conscious and progressive development. It has already been established that, in addition to laboratory tests, the results measured during field tests and match conditions are closer to the athletes' real performance; therefore, in our research, we preferred the use of field tests, as well (31, 33).

The objective of our study was to examine the effect of a 10-week intervention with the TOCA Football System tool and training method on elite youth athletes' sport-specific motor skills and anthropometric variables in a randomized controlled study.

## 2 Materials and methods

### 2.1 Selection and description of participants

In the spring of 2022, the U13-U14 junior football players of one of our country's elite academies in the Hungarian junior first division (NBI) are being examined. After eligibility assessment, 32 people were included in the study, which was randomly divided into two groups by www.randomizer.org, 15 people (13.25 years ± 0.58 years) made up the intervention group (hereafter TG) and 17 people (13.63 years ± 0.66 years) the control group (CG). The athletes included in the study all had more than five years of experience in football. They attended training sessions at an association football club six times a week, five of which were afternoon sessions, and one session was morning technical training. In the case of all participants, before the tests, the parents were provided information about the research, and they also had to fill out a written consent form. Exclusion criteria were defined as if the athlete suffered an injury during the previous two months or could not participate in at least 80% of the sessions.

### 2.2 Measurement tools

Measuring the physical ability of athletes is one of the most critical factors in modern football. Many tests are used to determine and analyze players' abilities and to examine the effectiveness of a training method (34, 35). To determine motor and physical performance, we used the tests of the measurement protocol preferred by the Hungarian Football Association (35), with which (31), also tested junior footballers. The body height of the tested sample was measured using the standard stadiometer technique, with their head in the Frankfort horizontal plane. We also determined the biological age of the examined sample. The calculation of morphological age can be summarized with the following formula:

MA=0.25∗(BHage+BWage+PLXage+CA±C(years)

where MA is morphological age, BH age is the age corresponding to the table value to which the subject's height is closest, BW age and PLX age are interpreted in the same way as for height, CA is calendar age, and C is any necessary correction (36, 37). A bioimpedance measurement method was used to measure body mass, muscle mass, and body fat percentage (InBody 770; InBody Co., Ltd., Seoul, South Korea).

#### 2.2.1 Yo-Yo intermittent recovery test level 1

The Yo-Yo Intermittent Recovery Test Level 1 (YYIR1) was used to determine the sport-specific endurance, maximum oxygen uptake capacity and maximum pulse values. Many studies deal with the examination of the physical condition of junior footballers (13–18 years old), the process of physiological adaptations that occur as a result of different training sessions, and the analysis of the physical performance achieved in matches to find out which variables can be used to predict the expected motor performance in matches (38–42). The YYIR1 is one of the most widely used, internationally accepted, and reliable field tests relevant to junior players, and it is a predictor of expected endurance performance in matches (40, 43–46).

A YYIR1 and a laboratory exercise test (Bruce protocol) were chosen based on Fang et al.'s research (47)—were also performed on the athletes to assess accurate physiological characteristics and to determine the expected maximum heart rate (HRmax) and estimated aerobic capacity (VO2max) (48):

VO2max=(Finaldistance(inmeters)×0.0084)+36.4

Based on the exercise tests, it can be established that the examination group was homogeneous regarding expected endurance performance (49) and maximum aerobic capacity (50, 51).

The Illinois Agility Test assessed multidimensional speed, agility, acceleration, change of direction speed and maneuverability (52–54).

#### 2.2.2 Illinois agility test

By performing the test, we determined the soccer players' agility, speed and deceleration ability, and ability to change direction by performing sequences of movements from different positions and angles. The players performed the survey in special soccer cleats. To measure performance, we used an infrared photocell gate (Fusion Sport Smartspeed, Australia), which was placed in a standard position at a height of 1 m at the start and finish lines. During the test, the test subjects start from a standing position behind the start line, and they start running when they hear the predetermined sound signal. The test subjects start sprinting from the start line; they run forward 10 m to run around a cone, then back 10 m to a cone placed at the height of the start line. Then, the test subjects weave in and out of the cones over a 10 m distance, complete a 180° turn and then weave back through the cones completing a 180° turn at the last cone. Then, the test subjects sprint forward 10 m to a cone, complete another 180° turn and sprint 10 m to the finish line. Finally, they cross the finish line by running between the infrared photocell gates placed at a height of 1 m (53, 55). The test subjects in the study had to complete the test as quickly as possible. Two options were provided to athletes to complete the course with a 3–5-min rest period between repetitions. During the statistical analysis, the fastest performance and the best time result were considered. The test was also performed by the players while dribbling a football.

### 2.3 Procedure

#### 2.3.1 Preintervention

For a start, the team staff was informed about the objectives of the study, and the research team ensured that parents signed their informed consent. Then, the research team studied and planned every training structure with the coaches, physical trainers, and both teams. After defining the groups, we ran pre-defined field tests and laboratory tests on the players in training week zero, and then after ten weeks, all the tests were repeated. The athletes performed the tests following the same order and with a minimum of 3 min and a maximum of 5 min of rest between tests. The tests, in both cases, were conducted indoors on synthetic turf at the Dárdai Pál Labdarúgó Akadémia Utánpótlás Edzőközpont (Senior Pál Dárdai Football Academy Youth Training Center) and the TOCA Football Center-Garami József Utánpótlás Labdarúgó Képző Központ (TOCA Football Center-József Garami Junior Football Training Center), at the constant temperature of 20°C–22°C.

#### 2.3.2 The intervention protocol

The TG *n* = 15 was determined randomly from the total sample *N* = 32. The CG was *n* = 17 people. During the entire period of the investigation (10 weeks), the morning training of the TG was held at TOCA Football Center-Garami József Utánpótlás Labdarúgó Képző Központ (TOCA Football Center-József Garami Junior Football Training Center) where they used the TOCA Football System (TOCA Football Inc. California Costa Mesa, US.) throughout their training sessions. Due to the championship schedule, the morning training sessions for both groups took place on Thursday between 07:00 and 08:15. The CG was instructed to continue the team's original training plan. In contrast, the TG group continued the same training plan using the soccer ball delivery system. Before training, the same warm-up protocol was used for both groups. Before the first session, the TG was verbally and visually informed about the operation of the new training device and the method. After the warm-up, the training continued according to the original training plan with the help of the device. During the training sessions, the main part after the warm-up lasted 45–50 min for each training session, with the same rest period added. The training sessions were conducted by the team coach and a trainer with a qualification in the TOCA Touch Trainer.

#### 2.3.3 Postintervention

After the intervention protocol, the TG and CG were evaluated at the same time of day as in the preintervention session, in a similar space with the same conditions.

### 2.4 Data analysis

For the treatment of the data, we used adequate statistical methods. Descriptive statistics were used to characterize the sample. Descriptive statistics are represented as mean ± standard deviation (SD) with standard mean difference data. Tests of normal distribution and homogeneity (Shapiro-Wilk's and Levene's, respectively) were conducted on all data before analysis. A paired sample *t*-test was used to determine differences as a repeated measures analysis (pre–post) within groups. When comparing normally distributed variables (anthropometry, Illinois agility test), we used a paired sample *T*-test where the significance level was set at *p* < 0.05. A non-parametric Wilcoxon test was used where the sample was not normally distributed (YOYO IR1 test- in the case of the TG), and the error limit was set at *p* < 0.05. The correlation coefficient (r) was the indicator of the effect size. To interpret the magnitude of the effect size, we adopted the following criteria: *r* = 0.10, small; *r* = 0.30, medium; *r* = 0.50, large. To discover between-group differences, a 2 (group: TG and CG) × 2 (time: pre, post) repeated-measures analysis of variance (mixed-design ANOVA) was calculated for each parameter. The delta percentage (Δ%) was calculated via the standard formula: Δ% = [(posttest score−pretest score)/pretest score] × 100. Partial eta-squared (*η*p2) effect sizes for the time × group interaction effects were calculated. An effect of *η*p2 ≥ 0.01 indicates a small, ≥0.059 a medium, and ≥0.138 a large effect, respectively (56). Data were analyzed using the IBM SPSS Statistics 27 software.

### 2.5 Ethical considerations

The study was conducted in accordance with the Declaration of Helsinki and approved by the University of Pécs Regional Research Ethics Committee (No. 9119-PTE 2022).

## 3 Results

### 3.1 The results of the anthropometric tests

In the case of BF%, no significant difference was found within either group. However, in the case of BM and SMM, we found a significant change within both groups (BM: *p* < 0.001, *r* = 0.804; *p* = 0.030 *r* = 0.511; SMM: *p* < 0.001, *r* = 0.812; *p* < 0.001, *r* = 0.716). Data showed that participants improved significantly after ten weeks in most anthropometric variables, except BF% within both groups Table 1.

**Table 1:** Anthropometric characteristics of the study sample before and after the 10-week intervention (mean ± SD).

	Young male football players (*N* = 32)							
	TG (*n* = 15)				CG (*n* = 17)			
	Pretest	Posttest	RM *t*-test (*p*)	Δ (%)	Pretest	Posttest	RM *t*-test (*p*)	Δ (%)
BH (cm)	163.2 ± 10.07	165.66 ± 9.2*	–	1.51	164.17 ± 12.19	166.11 ± 11.79*	–	1.18
BM (kg)	46.22 ± 8.6	48.19 ± 9.63*	*p* < 0.001 *r* = 0.804	4.26	50.4 ± 11.45	51.32 ± 11.37*	*p* = 0.030 *r* = 0.511	1.83
BF (%)	8.57 ± 4.01	8.59 ± 2.54	*p* = 0.988 *r* = 0.004	0.23	11.38 ± 4.94	10.79 ± 3.66	*p* = 0.288 *r* = 0.265	−5.18
SMM (kg)	23.05 ± 5.08	24.27 ± 5.27*	*p* < 0.001 *r* = 0.812	3.28	24.57 ± 6.62	25.25 ± 6.48*	*p* = 0.001 *r* = 0.716	2.77

BF%, body fat percentage; BH, body height; BM, body mass; SMM, skeletal muscle mass.

* significant differences (*p* < 0.05).

### 3.2 Results of the performance variables

The paired measures *t*-test with participant's performance variables (IAT, YYIR1, VO2max- in TG and CG) and Wilcoxon-test based on the variables normality test (YYIR1^post^ and VO2max^post^- in TG) that showed significant difference for all variables within groups (IAT without ball: *p* = 0.004, *r* = 0.675; IAT with ball: *p* = 0.002, *r* = 0.716; *p* = 0.023, *r* = 0.533; YYIR1: *p* = 0.001, *r* = 0.674, *p* = 0.004, *r* = 0.647) except of the IAT without the ball test within control group which showed no significant differences (IAT without ball: *p* = 0.675, *r* = 0.106). Data showed that participants improved significantly after ten weeks within groups in most performance variables, except IAT without the ball in CG. Absolute values for each variable at the pre- and post-test, along with the repeated measured ANOVA, showed no significant group x time interactions were observed between training groups (TG and CG) in any variable (*p* > 0.05) Table 2.

**Table 2:** Changes in the performance variables after the 10-weeks intervention (mean ± SD).

	Young male football players (*N* = 32)							
	TG (*n* = 15)				CG (*n* = 17)			
	pre	post	RM *t*-test (p)	Δ (%)	pre	post	RM *t*-test (p)	Δ (%)
IAT without ball (s)	15.82 ± 0.56	15.64 ± 0.59*	*p* = 0.004 *r* = 0.675	−1.14	15.85 ± 0.43	15.82 ± 0.42	*p* = 0.675 *r* = 0.106	−0.19
IAT with ball (s)	20.27 ± 1.14	19.92 ± 1.02*	*p* = 0.002 *r* = 0.716	−1.73	20.71 ± 1.14	20.40 ± 1.15*	*p* = 0.023 *r* = 0.533	−1.5
YYIR1. distance (m)	1,830.67 ± 333.71	2,073.33 ± 325.10*	*p* = 0.001 *r* = 0.674	13.26	1,435.29 ± 381.49	1,605.88 ± 381.49*	*p* = 0.004 *r* = 0.647	11.89
VO2max (ml/kg/min)	51.77 ± 2.8	53.81 ± 2.73*	*p* = 0.001 *r* = 0.674	3.94	48.45 ± 2.87	49.88 ± 3.20*	*p* = 0.004 *r* = 0.647	2.95
	ANOVA *p* values (*η*p^2^)							
	time		group			group × time		
IAT without ball (s)	*p* = 0.025, *η*^2^ = 0.157		*p* = 0.527, *η*^2^ = 0.013			*p* = 0.101, *η*^2^ = 0.087		
IAT with ball (s)	*p* < 0.000, *η*^2^ = 0.371		*p* = 0.254, *η*^2^ = 0.043			*p* = 0.807, *η*^2^ = 0.002		
YYIR1. distance (m)	*p* < 0.000, *η*^2^ = 0.550		*p* = 0.001, *η*^2^ = 0.311			*p* = 0.660, *η*^2^ = 0.007		
VO2max (ml/kg/min)	*p* < 0.000, *η*^2^ = 0.583		*p* = 0.001, *η*^2^ = 0.309			*p* = 0.348, *η*^2^ = 0.029		

RM, repeated measures (paired-samples *t*-test); IAT, Illinois agility test.

* significant differences (*p* < 0.05).

## 4 Discussion

This research aimed to examine the effect of the TOCA Football System on the body composition and motor performance (agility and sport-specific endurance) of junior athletes within the framework of a 10-week intervention program. When evaluating the results, we do not deal with the change in body height separately since it is more likely that it stems from age characteristics rather than from the training performed. The main findings of this study indicate that a TOCA Football training program in addition to regular training during the in-season period does not produce additional effects in anthropometric factors, sport-specific endurance and agility performance with the ball (dribbling) and without the ball in comparison with the control condition. This may indicate that the adaptations obtained during training with one TOCA training per week for ten weeks were similar to players participating in traditional soccer training. Related to these findings (57, 58) reported that regular participation in soccer-specific training sessions can be a sufficient incentive to increase the performance of soccer-specific skills.

However, based on the results of the body composition tests, it can be concluded that the average body mass of both groups changed significantly, in the case of the TG by +1.97 kg (*p* < 0.001) and in the case of the CG by +0.92 kg, (*p* = 0.030). The increase in muscle mass was primarily responsible for the increase in body mass in both groups since, in terms of body fat percentage, no significant difference was found in either group, while the body fat percentage of the TG increased by an average of +0.02%; (*p* = 0.988), while the body fat percentage of the CG increased by 0.59% on average; (*p* = 0.288). After the *t*-test, the results indicate that the effect of the intervention on body composition is not significant, as we found that the muscle mass (SMM) increased significantly in both groups. In the case of the TG with +1.22 kg (*p* < 0.001) and the CG with 0.68 kg (*p* = 0.001). However, observing the results, it can also be stated that although the increase in muscle mass can be statistically demonstrated in both cases, in the case of the TG, the muscle mass increased approximately twice as much. Csáki et al. (35) examined 76 people in the same age group and documented the body composition results of Hungarian junior soccer players over 12 months. At the end of the study, they experienced an average increase in muscle mass of +1.48 kg. So, while in a similar study such a change in muscle mass was only detectable in 12 months, with the TOCA training, it was achieved in only ten weeks. This can be supported by the results of a previous study by Szabó and Ács (26), in which TOCA and traditional football technical training, with the same training goal, were compared based on locomotor and physiological parameters that Polar Team Pro could measure. Based on their results, the athletes trained at a significantly higher intensity during the TOCA training sessions. This increased intensity—shown in the number of micromovements (number of stops, starts, changes of direction, sprints) and the distance covered in proportion to time—can benefit the increase in skeletal muscle mass, which is extremely important, since in football, as established by Mohr et al. (59), to carry out 30–40 acceleration phases and frequent jumps with a high efficiency level, one requires outstanding lower limb strength (60, 61). Previous studies have also reported that faster running speed is affected by the force applied to the ground, in addition to a higher step frequency (62–64). This physical axiom—according to which the greater the ground force (action), the greater the reaction force (reaction)—also proves the importance of skeletal muscle in improving the speed of footballers, which makes the athlete suitable for coping with the extreme physical demands experienced on the field of international soccer.

The agility tests used in football, including a combination of accelerations, decelerations, change of direction, explosiveness and turns, are the most relevant objective sport-specific tracks. Carrying out the aforementioned abilities with a high level of efficiency is decisive in terms of the effectiveness of the matches and the game situations. During our investigation, we performed the Illinois Agility tests with and without a ball. The paired sample *t*-test results show that in the case of the results of the TG, we experienced a significant improvement both in the agility test with a ball (*p* = 0.004) and in the agility test without a ball (*p* = 0.002), in the case of the CG we only found an improvement in the agility test with a ball (*p* = 0.023). In contrast, no significant difference was found in the agility test without a ball (*p* = 0.675). However, based on the result of the mixed-design ANOVA test, it can also be said that no significant group × time interaction were found during the agility tests comparison between the groups. This also means that even before the first examination, the intervention group members achieved better results than the control group. Another study conducted on eighteen youth soccer players that analyzed the effects of a 6-week coordination training intervention on physical fitness revealed no significant differences between the pre-and post-tests in agility performance (65). Based on these, it can be assumed that a longer intervention process would show a more accurate view regarding the development of differences between groups. The higher the number of repetitions and the more frequent the execution of tasks, the more micro-movements the athletes require, which is shown in the development of agility. Taylor et al. (66) found that during football matches, athletes experience some rhythm change every 3–4 s. This can be categorized with up to 1,200 acyclic movements, where 30–40 acceleration-deceleration phases and jumps (59), more than 700 changes of direction (67) and other intensive sport-specific techniques such as shooting, dribbling, tackles and fully body collision can be found (9). Based on these, it can be stated that success in football is largely determined by movement without the ball. Running performance is best characterized by frequent and fast sprints and continuous changes of direction, which is nearly 10% of the total amount of running performed in matches (68). In various 1–1 game situations, quick starts, decelerations, and direction changes can result in a 1–2 m advantage over the opponent, which can determine the success of a game situation. It is essential to consider that, during a match, more time is spent running without the ball than with the ball. In the case of offensive players, during the game situations before scoring a goal, an almost minimal number of (1–3) touches can be determined, which can be explained by the high-intensity rapid movements and direction changes performed in the preceding 3–5 s (69). Another advantage of the TOCA Football System could be that it forces the athletes to move significantly more without the ball (27).

An extensive analysis of football matches determined that players show different running performances in various phases of the match, where more robust phases are generally followed by weaker periods (59, 70). These analyzes and the obtained data confirm the need for the development of continuous and intermittent high-intensity physical performance in training (71, 72). In our study, we found that based on the results of the sport-specific endurance test (YYIR1), both groups' performance improved significantly.

During the follow-up measurements after the 10th week, in the TG, the athletes ran an average of 242.66 m more (*p* = 0.001), while in the CG, they ran an average of 170.59 m more (*p* = 0.004). Based on these, it can be concluded that both groups' YYIR1 performance and oxygen uptake capacity improved significantly (*p* = 0.001) and (*p* = 0.004) during the ten weeks. Based on the mixed-design ANOVA test, the difference between the averages of the two groups shows no significant group × time interactions, but that the results of the TG were slightly better, which can also be explained by the higher intensity of the TOCA training (26), which is also reflected in the increase in muscle mass, micromovements without the ball and endurance indicators. This can also be explained by (57, 58) with the findings that regular participation in soccer-specific training can be a sufficient incentive to increase the performance of soccer-specific skills.

An analysis of VO2 max, which is regarded as one of the most critical components of endurance performance (73). The mean VO2max of elite soccer players generally ranges from 55 to 68 ml/kg/min (38), much higher than the values we measured, which can be between 49.9 and 53.8 ml/kg/min. Recently, at another Hungarian academy, the endurance parameters of youth soccer players were also examined, where a value of 57.6 ml/kg/min was measured, which is also higher than what we experienced (37).

Regarding the research, the tests were carried out in an age group that is a sensitive age for motor skill learning. At this age, the development of the bone and muscle system progresses by leaps and bounds, affecting adolescents' physical performance. This partially explains why the body height and body mass of the sample increased significantly until the 10-week intervention (BH: *p* < 0.001, *p* < 0.001; BM: *p* < 0.001, *r* = 0.804; *p* = 0.030 *r* = 0.511). Among the most critical morphological changes that can be observed at this time are the accelerated change in body sizes and internal organs, changes in body proportions, and changes in body composition. These small changes can affect performance and motor coordination. Through the quantitative and qualitative development of muscles, their strength improves significantly. They become not only stronger but their training load can also be increased. In boys, the absolute mass of the body fat can also decrease (74). During puberty, the favorable conditions for motor skill learning are temporarily reduced. The adolescent child's motor coordination deteriorates and becomes impaired, and previously successfully mastered movements may appear clumsy. These are usually related to the intense increase in body length dimensions typical of adolescence. In the case of adolescent athletes, the duration of motor skill learning and the number of repetitions of the exercises must be chosen carefully and according to a plan (75). Bearing in mind the above, it can be stated that training should aim not only to increase performance but also to maintain motor coordination for the affected age group. According to Dubecz (30) and Harsányi (29), the performance of athletes in the affected age group can also start to deteriorate due to the inappropriate use of training stimuli in training sessions. Furthermore, it can also be established based on own research and given the characteristics of soccer, implementing mixed approaches in the training process, as well as introducing strength and power training, for U14 players is of paramount importance to build more resilient athletes (76–78).

## 5 Conclusions

Overall, it can be concluded that the integration of the TOCA Football System into football training is safe, even for an age group that is sensitive to motor skill learning (U13-U14). The results confirmed that using the TOCA Football System does not negatively affect the athletes' performance in terms of the measured parameters. In the case of the sample we examined, we experienced a significant improvement in most of the parameters within groups. However, the comparison between the groups did not show a significant difference, so a positive interaction cannot be established during the intervention. It was also found that it is worth approaching the examination of football-related motor skills in a more complex manner and supplementing the research with other tests, such as sprint tests, tests measuring the dynamic strength of the lower limb, or sports psychology tests.

## 6 Limitations

The main limitation of our study is that we could get a more accurate view of the long-term effect of the tool if the study was carried out with a larger number of elements over a longer intervention period, including other age groups. We could also get a more accurate view of the athletes' estimated aerobic capacity if laboratory tests were used. In the present study, we only examined junior male athletes, so we could get more reliable results if the research were repeated with the involvement of female athletes.

## Data Availability

The raw data supporting the conclusions of this article will be made available by the authors, without undue reservation.
